# Incidence, risk factors, and viral etiology of community-acquired acute lower respiratory tract infection among older adults in rural north India

**DOI:** 10.7189/jogh.11.04027

**Published:** 2021-04-03

**Authors:** Rakesh Kumar, Lalit Dar, Ritvik Amarchand, Siddhartha Saha, Kathryn E Lafond, Debjani R Purakayastha, Ramesh Kumar, Avinash Choudekar, Giridara Gopal, Shivram Dhakad, Venkatesh Vinayak Narayan, Abhishek Wahi, Reshmi Chhokar, Stephen Lindstrom, Brett Whitaker, Aashish Choudhary, A B Dey, Anand Krishnan

**Affiliations:** 1 All India Institute of Medical Sciences, New Delhi; 2 Influenza Division, Centers for Disease Control and Prevention- India Country Office, New Delhi, India; 3 Centers for Disease Control and Prevention, Atlanta, Georgia, USA

## Abstract

**Background:**

There are limited data on incidence, risk factors and etiology of acute lower respiratory tract infection (LRTI) among older adults in low- and middle-income countries.

**Methods:**

We established a cohort of community dwelling older adults ≥60 years and conducted weekly follow-up for acute respiratory infections (ARI) during 2015-2017. Nurses assessed ARI cases for LRTI, collecting combined nasal/throat swabs from all LRTI cases and an equal number of age- and sex-matched asymptomatic neighbourhood controls. Swabs were tested for influenza viruses, respiratory syncytial virus (RSV), human metapneumovirus (hMPV), and parainfluenza viruses (PIV) using polymerase chain reaction. LRTI and virus-specific LRTI incidence was calculated per 1000 person-years. We estimated adjusted incidence rate ratios (IRR) for risk factors using Poisson regression and calculated etiologic fractions (EF) using adjusted odds ratios for detection of viral pathogens in LRTI cases vs controls.

**Results:**

We followed 1403 older adults for 2441 person-years. LRTI and LRTI-associated hospitalization incidences were 248.3 (95% confidence interval (CI) = 229.3-268.8) and 12.7 (95% CI = 8.9-18.1) per 1000 person-years. Persons with pre-existing chronic bronchitis as compared to those without (incidence rate ratio (IRR) = 4.7, 95% CI = 3.9-5.6); aged 65-74 years (IRR = 1.6, 95% CI = 1.3-2.0) and ≥75 years (IRR = 1.8, 95% CI = 1.4-2.4) as compared to 60-64 years; and persons in poorest wealth quintile (IRR = 1.4, 95% CI = 1.1*-*1.8); as compared to those in wealthiest quintile were at higher risk for LRTI. Virus was detected in 10.1% of LRTI cases, most commonly influenza (3.8%) and RSV (3.0%). EF for RSV and influenza virus was 83.9% and 83.6%, respectively.

**Conclusion:**

In this rural cohort of older adults, the incidence of LRTI was substantial. Chronic bronchitis was an important risk factor; influenza virus and RSV were major viral pathogens.

Acute lower respiratory tract infections (LRTI) like pneumonia, acute bronchitis and acute bronchiolitis are an important cause of morbidity and mortality among older adults globally [1]; studies have reported high incidence of LRTI among older adults [2,3]. Pneumonia has been associated with high rates of hospitalization among older adults [4], and an increasing trend in hospitalization due to pneumonia in this age group over the last few decades has been documented [5]. A meta-analysis estimated there were 6.8 million pneumonia-associated hospitalizations among older adults worldwide in the year 2015 with 1.1 million in-hospital deaths [4]. Studies have reported substantial 30-day mortality of up to 12%-14% among older adults after an episode of pneumonia [6,7]. Pneumonia among older adults imposes a significant economic burden; one study reported the annual cost of pneumonia among adults in the United States to be more than$ 17 billion annually in 2005 [8]. Other studies have also reported high costs of pneumonia care among older adults [9,10].

In India, community-acquired LRTI is a common cause of death among older adults [11]. Studies conducted to date in India on etiology of LRTI among older adults were hospital-based and focused on bacterial etiology [12,13]. As many patients in this age group have poor access to hospitals [14], hospital-based studies are likely to give a biased estimate of the incidence and etiology of LRTI. Viruses are implicated in a large number of LRTI in this age group. [15]. Information on burden and viral etiology of LRTI among older adults in India is lacking. In recent systematic reviews on community-acquired pneumonia-associated hospitalization or on viral etiologic agents of acute respiratory infections (ARI) among older adults, none of the included studies were from India [4,15].

As the proportion of older adults increase with increasing life-expectancy in India, LRTI among older adults is likely to become a significant public health problem. The National Programme for the Healthcare of Elderly (NPHCE) was launched in India to provide comprehensive preventive care and health promotion to older adults [16]. Though vaccination against pneumococcus and influenza are recommended by the World Health Organization, lack of robust estimates on burden and etiology of LRTI in India hampers effective policy formulation in this regard. To our knowledge, no study has estimated the burden of LRTI among community-dwelling older adults through longitudinal follow-up in India. In 2017, India had 13% of the global population of individuals aged 60 years and above, therefore any robust global estimate of burden of LRTI needs data from India. Hence, we established a dynamic cohort of community-dwelling older adults (≥60 years of age) in rural north India to generate evidence on incidence and viral etiology of LRTI.

## METHODS

We established a dynamic cohort in five villages, four rural and one peri-urban, in the Ballabgarh block in the Faridabad district of the northern Indian state of Haryana. We conduct a census every year to update the demographic details of this population. The study area had a total population of 17 966 as of 1 January 2015. The geographic coordinates of the study area are 28.3388°N, 77.3206°E. There are three distinct seasons ie, winter (November-February), summer (March-June) and monsoon (July-October). Average annual rainfall is 657 mm. Healthcare in the study area is mostly delivered in clinics by unqualified private health practitioners and at three public health sub-centres managed by auxiliary nurse-midwives. The study setting has been described previously [17].

We generated a list of individuals from the demographic database who have been residing in the study area for at least six months and who were age ≥60 years on 1 January 2015. Field investigators approached those eligible at home to obtain written informed consent for participation. Field investigators continued to enrol resident individuals as they turned 60 during the study period. We excluded those individuals who could not give valid informed consent, were not likely to be available for surveillance during the period of study for any reason, or refused to be part of the cohort. Participants exited the cohort if they migrated out of the study area, died, or withdrew consent to participate.

Project physicians screened the participants for pre-existing chronic bronchitis and other chronic morbidities including self-reported diabetes, hypertension, cardiac disorders, stroke, liver disorders, renal disorders and malignancy through medical history and clinical examination at baseline. The clinical examination consisted of systemic examination of the participant by a physician including pulse and blood pressure measurement, and auscultation and was used to diagnose condition like hypertension, paralysis, hepatomegaly etc. Chronic bronchitis was defined as presence of cough for at least 3 consecutive months for 2 successive years [18]. Information on socio-economic indicators like possession of household items, use of solid fuel in the household, and individual risk factors like co-morbidities and smoking habits were collected at the baseline interview using a structured questionnaire.

We followed all participants through weekly surveillance for acute respiratory infections (ARI) at home by trained field investigators. ARI was identified based on new onset of either cough, nasal discharge, sore throat, or difficulty in breathing; or worsening of any pre-existing respiratory symptoms (cough, difficulty in breathing, increase in sputum production). In those with chronic bronchitis, definition was modified to include new onset of fever in past 7 days. Trained nurses visited all the ARI cases detected on the same day for clinical assessment. They classified ARI cases as either LRTI, or in those without symptoms and signs suggestive of LRTI as acute upper respiratory tract infection (URTI). LRTI was defined as ARI with respiratory rate ≥20/min, and presence of ≥1 of the lower respiratory symptoms of productive cough, chest pain on breathing, or wheezing [19]. Surveillance case definitions are given in **Box 1**. Nurses recruited asymptomatic controls without symptoms of ARI in the previous 7 days in the ratio of 1:1 for every case of LRTI. Controls were matched for age (±5 years), sex, and neighbourhood. All those hospitalized were either visited in the hospital or at home, and the discharge summary was accessed to ascertain diagnosis at admission.

Box 1 **Surveillance definitions used in the study.**

Chronic bronchitis: Presence of cough for at least 3 consecutive months for 2 successive years.

Acute Respiratory Infection (in those without chronic bronchitis): New onset of either cough, nasal discharge, sore throat, or difficulty in breathing in past 7 days.

Acute Respiratory Infection (in those with chronic bronchitis): Worsening of any pre-existing respiratory symptoms (cough, difficulty in breathing, increase in sputum production), or new onset of fever or any of following respiratory symptoms, ie, cough, nasal discharge, sore throat, sputum production, or difficulty in breathing in past 7 days.

Acute Lower Respiratory Tract Infections: Cases of ARI with respiratory rate ≥20/min, and ≥1 lower respiratory symptom of productive cough, chest pain on breathing, or wheezing.

Acute Upper Respiratory Tract Infection: Cases of ARI without symptoms and signs suggestive of pneumonia.

Mid-turbinate nasal and throat specimens were obtained from all LRTI cases and asymptomatic controls. Specimens were collected using Dacron swabs, combined in the same tube of viral transport medium and transported to the virology laboratory at the All India Institute of Medical Sciences (AIIMS), New Delhi on ice in triple-sealed containers within 24 hours of collection. Specimen aliquots were stored at -80°C until testing was initiated. Total nucleic acid was extracted from each specimen with the Roche LC 2.0 TNA extractor platform using the Roche MagNa Pure LC 2.0 Total Nucleic Acid Isolation kit (Roche Inc., Germany). Real-time reverse transcription polymerase chain reaction (rRT-PCR) was performed using United States Centers for Disease Control and Prevention (CDC) protocols for detection of influenza viruses, respiratory syncytial virus (RSV), human metapneumovirus (hMPV), and parainfluenza viruses 1-3 (PIV) [20,21].

We developed standardized protocols for paper-based data collection, interviewing, taking medical measurements, collecting biological specimens, and processing the collected data. Study staff were trained and re-trained in these protocols. Senior study staff supervised field data collection. The virology laboratory followed appropriate internal and external quality assurance protocols.

Data were entered into a MySQL database, and encrypted data were regularly backed up. A new episode of ARI or LRTI was defined as presence of new symptoms after a symptom-free interval of 14 days. All weekly data on symptoms for each participant were linked to delineate each episode of ARI. Person-time (person-year) of risk was used as the denominator for the estimation of incidence of LRTI and hospitalization with 95% confidence intervals (95% CIs) using the normal approximation method.

We calculated incidence rates and crude rate ratios stratified for sub-categories of age, sex, presence of co-morbidities, ever use of smoked tobacco, wealth score quintile, and use of solid fuel in the household. Age was recorded as the age at the start of the episode. We calculated wealth score at household level through principal components analysis of data on possession of household items. We calculated adjusted incidence rate ratios (aIRR) for these risk factors through multi-level Poisson regression modelling, adjusting for above-listed risk factors at the individual level, as well as clustering at the household level. We calculated prevalence of each pathogen as the proportion of LRTI episodes associated with the specific pathogen out of all LRTI episodes detected among the participants. We constructed multivariable logistic regression models for each pathogen to estimate the adjusted odds ratio (aOR) with the dependent variable in each model being the clinical status, ie, LRTI case or asymptomatic control. For calculation of aOR, presence of a virus was considered as the exposure; we compared odds of a positive detection of a specific virus between LRTI cases and asymptomatic controls, and adjusted for confounders defined a priori (age, sex, and month of sample collection). We used the aOR to calculate the pathogen-specific etiologic fraction (EF) or the proportion of LRTI in the population with a viral pathogen in which the pathogen can be attributed to have played a possible causal role in disease development using the equation EF = [aOR–1]/aOR.This approach has previously been used to calculate etiologic fraction of viruses in LRTI [15,22,23]. Agent-specific crude LRTI incidence rates were multiplied by EF to calculate adjusted LRTI incidence rates. All analyses were conducted in Stata 12 (StataCorp, 2011). The study was approved by the institute ethics committee of All India Institute of Medical Sciences (AIIMS), New Delhi (Ethics approval No: IEC/OP-07/14.11.2014, RP-50/2014) and the Institutional Review Board at United States Centers for Disease Control and Prevention (US CDC), Atlanta (protocol number-6296).

## RESULTS

**Figure 1:**
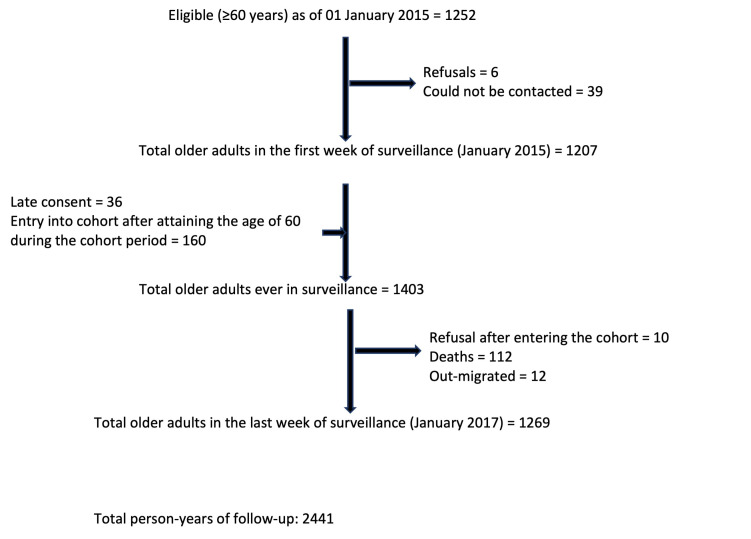
Flowchart depicting recruitment and loss to follow up of the study participants of the cohort.

We followed 1403 older adults from 979 households during January 2015 to January 2017 for a total of 2441 person-years (**Figure 1**). One hundred and twelve participants died during the period of follow up, and 12 migrated out of the study area. Of the 1403, 53% were women, 25.4% had pre-existing chronic bronchitis, 42.3% were currently smoking tobacco, and 59.7% had ever smoked tobacco. Seventy percent of the households were using solid fuel. A total of 8834 episodes of ARI and 606 episodes of LRTI were detected during the study period. One or more of the 606 LRTI episodes occurred among 371 participants. Among 371 participants with LRTI, 66 had two episodes, 40 had three episodes, 10 had four episodes, 11 had five episodes and 3 had six episodes during the study period. Among participants with LRTI, the mean respiratory rate was 28.2 (SD = 4.9); 99.3% reported cough 93.4% of which was productive, 45.4% reported difficulty in breathing, 47.4% reported fever, 61.1% reported chest pain, and 61.7% reported wheeze. All three lower respiratory symptoms of productive cough, chest pain and wheeze was reported in 41.6% of participants with LRTI.

The unadjusted incidence of LRTI per 1000 person-years was 248.3 (95% CI = 229.3-268.8); 291.9 (95% CI = 262-325.3) among men and 211.0 (95% CI = 187.6-237.3) among women. Incidence of LRTI increased with age among both men and women. Incidence of LRTI per 1000 person-years among those in the age group 60-64 years was 144.7 (95% CI = 122.5-171.0) compared to 375.1 (95% CI = 325.2-432.8) among those aged ≥75 years (**Table 1**). There were 31 LRTI hospitalization episodes. The LRTI hospitalization rate per 1000 person-years was 12.7 (95% CI = 8.9-18.1): 16.9 (95% CI = 10.8-26.6) among men and 9.1 (95% CI = 5.2-16.1) among women. Sex-specific hospitalization rates increased with age in men but not in women. Thirty-day all-cause mortality after an episode of LRTI was 3.5% with 21 deaths in 606 episodes.

**Table 1:** Age and sex-specific incidence of LRTI and LRTI -associated hospitalization among adults age ≥60 y in rural Ballabgarh, India

Age group (years)	Total person years of follow up	Total episodes of LRTI	Incidence of LRTI*(95% CI)			Incidence of LRTI-associated hospitalization (95% CI)*		
**Men**	**Women**	**Combined**	**Men**	**Women**	**Combined**
60-64	953.6	138	141.1 (110.0-181.0)	147.8 (118.0-185.0)	144.7 (122.5-171.0)	9.1 (3.4-24.4)	11.7 (5.3-26.1)	10.5 (5.6-19.5)
65-74	986.2	280	345.9 (295.7-404.7)	231.7 (194.3-276.2)	283.9 (252.5-319.2)	15.5 (7.4-32.6)	9.3 (3.9-22.4)	12.2 (6.9-21.4)
≥75	501.2	188	471.3 (391.0-568.1)	291.3 (233.3-363.7)	375.1 (325.2-432.8)	34.3 (17.1-68.5)	3.7 (0.5-26.6)	18.0 (9.3-34.5)
Total	2441.0	606	291.9 (262.0-325.3)	211.0 (187.6-237.3)	248.3 (229.3-268.8)	16.9 (10.8-26.6)	9.1 (5.2-16.1)	12.7 (8.9-18.1)

LRTI – lower respiratory tract infection, CI – confidence interval

*Per 1000 person-years.

Persons with pre-existing chronic bronchitis as compared to those without (IRR = 4.7, 95% CI = 3.9-5.6); aged 65-74 years (IRR = 1.6, 95% CI = 1.3-2.0) and ≥75 years (IRR = 1.8, 95% CI = 1.4-2.4) as compared to 60-64 years; and persons in poorest wealth quintile (IRR = 1.4, 95% CI = 1.1*-*1.8) as compared to wealthiest quintile were at higher risk for LRTI (**Table 2**).

**Table 2:** Risk factors for LRTI among adults age ≥60 years in rural Ballabgarh, India

Risk factor	Incidence rate* (95% CI)	Crude rate ratio (95% CI)	Adjusted rate ratio (95% CI)
**Age (years):**			
60-64 (n = 582)	144.7 (122.5-171.0)	Reference	Reference
65-74 (n = 536)	283.9 (252.5-319.2)	1.9 (1.6-2.4)	1.6 (1.3-2.0)
≥75 (n = 285)	375.1 (325.2-432.8)	2.6 (2.1-3.2)	1.8 (1.4-2.4)
**Sex**			
Male (n = 659)	291.9 (262.0-325.3)	Reference	Reference
Female (n = 744)	211.0 (187.6-237.3)	0.7 (0.6-0.8)	0.9 (0.7-1.1)
**Chronic bronchitis**			
No (n = 1047)	123.8 (108.8-141.0)	Reference	Reference
Yes (n = 356)	630.2 (569.8-697.0)	5.1 (4.3-5.9)	4.7 (3.9-5.6)
**Any other co-morbidity^†^**			
No (n = 1232)	239.5 (219.6-261.2)	Reference	Reference
Yes (n = 171)	308.0 (252.1-376.2)	1.3 (1.0-1.6)	1.3 (1.1-1.7)
**Current use of tobacco smoke**§			
No (n = 423)	173.7(146.9-205.3)	Reference	Reference
Yes (n = 636)	290.7 (261.5-323.0)	1.7(1.4-2.0)	1.1 (0.9-1.4)
**Wealth quintile**¶			
First (Poorest) (n = 282)	311.3 (265.4-365.2)	1.5(1.2-1.8)	1.4 (1.1-1.8)
Second (n = 280)	283.6 (240.4-334.5)	1.3(1.0-1.7)	1.2 (1.0-1.6)
Third (n = 279)	229.3 (189.9-276.9)	1.1 (0.8-1.4)	1.0 (0.7-1.3)
Fourth (n = 285)	206.8 (170.7-250.7)	1.0 (0.7-1.3)	0.8(0.6-1.2)
Fifth (Wealthiest) (n = 275)	211.6 (174.3-256.9)	Reference	Reference
**Use of solid fuel in the household**¶			
No (n = 291)	219.0 (187.0-256.4)	Reference	Reference
Yes (n = 687)	260.6 (237.6-285.7)	1.2 (1.0-1.4)	1.1 (0.9-1.4)

LRTI – lower respiratory tract infection, CI – confidence interval

*Per 1000 person-years

†Any other co-morbidity included self-reported diabetes, hypertension, cardiac disorders, stroke, liver disorders, renal disorders and malignancy.

§Data on tobacco smoke was not available for some of the participants.

¶Household level data not available from two participants from one household.

**Figure 2:**
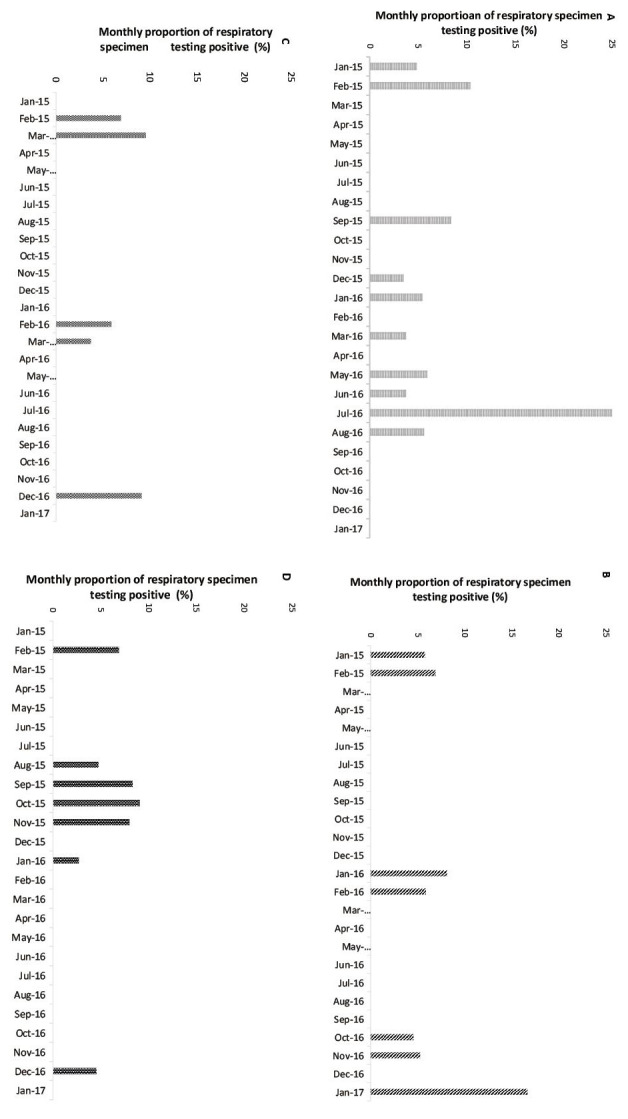
Monthly detection of respiratory viruses among adults age ≥60 years with acute lower respiratory tract infections (LRTI) in rural Ballabgarh, India, January 2015-January 2017. **Panel A.** Influenza viruses. **Panel B.** Respiratory syncytial virus. **Panel C.** Human meta-pneumovirus. **Panel D.** Parainfluenza viruses.

Viral pathogens were detected in 61 (10.1%) of the LRTI cases. The viruses detected among LRTI cases were influenza (23, 3.8%), RSV (18, 3.0%), hMPV (9, 1.5%), and PIV (11, 1.9%). Adjusted odds ratio for influenza viruses was 6.1 (95% CI = 2.3-16.6) and for RSV was 6.2 (95% CI = 2.0-19.1). Adjusted etiologic fractions for influenza viruses and RSV were 83.6% (95% CI = 56.5%-94.0%) and 83.9% (95% CI = 50.0%-94.8%) (**Table 3**). The adjusted incidence per 1000 person-years varied from 2.2 (hMPV) to 7.9 (influenza viruses). While the incidence of influenza, RSV, and hMPV-associated LRTI was higher in older age groups, PIVs were more common in younger age groups (**Table 4**). The detection of all viral pathogens were more common during the winter months (November-February), while influenza viruses and PIVs also circulated during the monsoon season (July-October) (**Figure 2**).

**Table3:** Etiologic fraction for viral pathogens associated with LRTI among adults age ≥60 years in rural Ballabgarh, India

Pathogen	Total detections among 606 LRTI cases	Positivity among LRTI cases, % (95% CI)	Positivity among 780 controls, % (95% CI)	Crude OR (95% CI)	aOR† (95% CI)	Adjusted etiologic fraction§, % (95% CI)	Adjusted positivity among LRTI cases¶, % (95% CI)
Influenza viruses	23	3.8 (2.3-5.3)	0.6 (0.1-1.2)	6.1 (2.2-20.7)	6.1 (2.3-16.6)	83.6 (56.5-94.0)	3.2 (1.3-5.0)
Respiratory syncytial virus (RSV)	18	3.0 (1.6-4.3)	0.5 (0-1.0)	5.9 (1.9-24.2)	6.2 (2.0-19.1)	83.9 (50.0-94.8)	2.5 (0.8-4.1)
Human metapneumo virus (hMPV)	9	1.5 (0.5-2.4)	0.6 (0.1-1.2)	2.3 (0.7-8.9)	2.5 (0.8-7.8)	60.0 (0-87.2)	0.9 (0-2.1)
Parainfluenza viruses (PIV)	11	1.9 (0.7-2.9)	0.6 (0.1-1.2)	3.1 (1.0-11.4)	4.4 (1.5-12.8)	77.3 (33.3-92.2)	1.5 (0.2-2.7)

LTRI – lower respiratory tract infection, CI – confidence interval, OR – odds ratio, aOR – adjusted odds ratio

*Per 1000 person-years.

†Adjusted for age, sex, and month of infection.

§Calculated by the formula 1 – (1/aOR) × 100 and denotes proportion of LRTI cases among those with viral detection that can be attributed to the viral pathogen.

¶Calculated by multiplying the positivity of viral pathogens among LRTI cases with respective adjusted etiologic fractions and denotes proportion of LRTI in the population with a viral pathogen in which the pathogen can be attributed to have played a possible causal role in disease development.

**Table 4:** Adjusted incidence of virus-associated LRTI among adults age ≥60 years in rural Ballabgarh, India

Age group (years)	Adjusted incidence of virus-associated LRTI* (95% CI)†			
**Influenza viruses**	**Respiratory syncytial virus**	**Human metapneumovirus**	**Parainfluenza viruses**
60-64	5.3 (1.6-13.2)	2.6 (0.5-8.1)	0.6 (0-6.5)	4.9 (0.9-12.9)
65-74	8.4 (3.1-17.7)	7.6 (2.4-14.7)	2.5 (0-9.4)	3.1 (0.5-10.0)
≥75	11.7 (3.8-27.5)	10.1 (2.7-22.3)	4.8 (0-18.6)	3.1 (0.3-14.8)
Total	7.9 (3.6-13.3)	6.2 (2.3-9.8)	2.2(0-6.2)	3.8 (0.9-8.0)

CI – confidence interval

*Per 1000 person-years.

†Adjusted by multiplying the crude incidence of virus associated LRTI by respective etiologic fractions.

## DISCUSSION

This study estimated the incidence, risk factors and etiology of LRTI among community-dwelling older adults in India through a multi-year longitudinal follow up. We found the incidence of LRTI in older adults to be 248 per 1000 person-years, while incidence of LRTI hospitalization was 12 per 1000 person-years. Those with pre-existing chronic bronchitis were five times more likely than those without pre-existing chronic bronchitis to acquire LRTI during the study period. Those aged 75 years or more, or in the poorest wealth quintile were also at increased risk of LRTI. A virus could be detected only in 11% of the LRTI episodes; influenza viruses being most common. Thirty-day all-cause mortality after an episode of LRTI was 3.5%.

We found an incidence of LRTI of 248.3 per 1000 person-years, which is similar to the estimate from the Global Burden of Disease (GBD) study for older adults in South Asia. The GBD study estimated incidence of LRTI in individuals >70 years to be 230.7 per 1000 person-years (95% CI = 214.9-247.7) [24]. We could not find any comparable data on LRTI or pneumonia incidence from India or other developing countries. A population-based study in United Kingdom reported an incidence of LRTI ranging from 92.2 episodes per 1000 person-years in 65-69 years to 187.9 per 1000 person years in 85-89 years. This study also reported the incidence of community-acquired pneumonia as 2.8-21.8 per 1000 person-years [25]. Our study was community-based and used a symptom complex of high respiratory rate along with one of the three symptoms: productive cough, chest pain, and wheezing. High respiratory rate has been shown to have a high diagnostic odds ratio and specificity to diagnose radiographic pneumonia in adults and is unlikely to overestimate the burden of LRTI in our setting [26].Our study like other studies also reports a much higher incidence of LRTI with increasing age. LRTI incidence was two and half times greater among those ≥75 years (375.1 per 1000 person-years) as compared to those in the 60-64-year age group (144.7 per 1000 person-years). LRTI incidence was higher among men (291.9 per 1000 person-years) than women (211.0 per 1000 person-years). This higher incidence in men occurred in the two older age groups that we studied but not in those 60-64 years.

The overall incidence of LRTI-associated hospitalization in our study was 12.7 per 1000 person-years. Our observed incidence rate of hospitalization of 12.2 per 1000 person-years (95% CI = 6.9- 21.4) in older adults aged 65-74 years is higher than a previous meta-analysis, which had reported the rate of pneumonia-associated hospitalization in developing countries to be 4.9 per 1000 person-years in the same age group [4]. However, our observed incidence rate is comparable to the findings of studies conducted in Japan (65-74 years, 16.9 per 1000 person-years) [27] and US (65-79 years, 6.3 per 1000 person-years) [28] between 2010 and 2013. A hospital based study conducted in Vietnam between 2009 and 2010 found a much lower incidence of pneumonia per 1000 person-years of 2.67 in the age group 65-74 and 6.95 in those ≥75 years [29]. We found higher incidence rates of hospitalization among those aged ≥75 years (18.0 per 1000 person years, 95% CI = 9.3-34.5) which was similar to the estimated incidence reported in the meta-analysis for developing countries (75-84 years, 14.6 per 1000 person-years; >85 years, 5.6 per 1000 person-years) [4] and rates in US (16.4 per 1000 person-years in those ≥80 years) [28]; however, this was lower than what was observed in Japan (75.4 per 1000 person-years in those ≥85 years) [27]. Thirty-day mortality after an episode of LRTI in our study was 3.5%, which was lower than other settings, [27,30] though similar mortality was found among ambulatory cases in a few studies in Europe [6,7].

Our study found higher age, pre-existing chronic bronchitis, other pre-existing co-morbidities, and being in the poorest household income quintile as risk factors for LRTI. The high incidence of LRTI in our cohort could possibly be due to a high prevalence of pre-existing chronic bronchitis; one in four participants suffering from the condition. Previous studies have reported chronic bronchitis and smoking as independent risk factors for LRTI [7,29,31]. The large number of households using solid fuel indoors and high prevalence of smoking in the study population might have contributed to the high burden of chronic bronchitis and LRTI, though we could not find a positive association between LRTI incidence and solid fuel use in the household or smoking.

In our study, viral pathogens could be detected from 10.1% of the cases of LRTI. Influenza viruses and RSV were the most commonly detected. To our knowledge, no previous study has reported the detection of viral pathogens in community-acquired LRTI among older adults in India. Our findings are similar to another study from China, which reported detection of viral pathogens in 14.7% hospitalized LRTI cases among those ≥65 years; influenza being the most common pathogen [32]. This study also reported lower detection of viral pathogens with increasing age. In studies conducted in other countries in Asia-Pacific region, viral pathogens could be detected in 15%-29% of cases of pneumonia among older adults; rhinoviruses (HRV) and influenza viruses were the most commonly detected viruses in those studies [27,29,33]. In a study conducted in the United States, HRV was the most common viral pathogen detected from hospitalized pneumonia cases, followed by influenza viruses and hMPV.^28^ Others have reported influenza viruses and RSV as the most common viral pathogens in pneumonia in older adults [34]. A meta-analysis that assessed the etiologic role of respiratory viruses in ARI in older adults found high etiologic fractions for RSV (odds ratio (OR) = 8.5; EF = 88%), influenza viruses (OR = 8.3; EF = 88%), and hMPV (OR = 9.8; EF: 90%) [15]. This is similar to our study, except for hMPV, which had a lower etiologic fraction.

The GBD study estimated the burden of influenza and RSV per 1000 persons among adults of age ≥70 years [1]. We found a similar incidence of RSV-associated LRTI. The incidence of influenza-associated pneumonia was lower in our study; though similar estimates for influenza-associated pneumonia were reported by another study from Japan [27]. The observed seasonality of influenza virus transmission was similar to that found in other settings and age groups in India [35].

There are some limitations to our studies. The incidence of LRTI must be interpreted with caution, as the diagnosis was based on a reported syndrome and confirmation was not done through radiography. The use of sensitive case definition in absence of radiography could have also led to inclusion of non-infective aggravations of chronic respiratory diseases. However, guidelines of the British Thoracic Society have advocated diagnosis in the community based predominantly on clinical symptoms [36]. Testing for only four viral pathogens and not testing for HRV could be the reason for lower viral detection among LRTI cases in our study. Since specimen collection visits took place once a week, we may have underestimated viral burden as viral shedding might have ceased by the time study staff arrived at the household. There may be issues about attributing causality for LRTI from detected pathogens from upper respiratory specimens, as they may simply indicate carriage and not infection, though we attempted to account for this by using etiologic fraction analysis.

Nevertheless, our study is probably the first such community-based cohort study of older adults studying etiology of LRTI in India and one of the few from a low-resource setting. The high burden of LRTI in this population and associated mortality may require multi-modal interventions including vaccination against pathogens including influenza; support for smoking cessation for management of chronic bronchitis; and appropriate management of co-existing morbidities. In India, currently there is no public health vaccination program for older adults, and only limited influenza or pneumococcal vaccination use in the private sector for older adults. More such community-based studies are required to estimate the disease and economic burden of LRTI among older adults to inform the policy makers. India’s National Programme for the Healthcare of Elderly provides an ideal platform for introduction of these interventions in the country.

## CONCLUSIONS

The incidence of clinically-defined LRTI was 248 per 1000 person-years among older adults in rural India in this rural cohort of community-dwelling older adults with incidence increasing with age. Influenza viruses and RSV were the most common viral pathogens detected. These results suggest the need for a comprehensive multi-pronged population-based approach for LRTI prevention and control among the elderly.
